# Supplementary material for: Tau PET correlates with different Alzheimer’s disease‐related features compared to CSF and plasma p‐tau biomarkers
Source: EMBO Mol Med. 2021 Jul 13;13(8):e14398. doi: 10.15252/emmm.202114398 (PMC8350902; doi:10.15252/emmm.202114398)
Supplement: Supplementary file 1 — Appendix [file EMMM-13-e14398-s003.pdf]

SUPPLEMENTARY MATERIAL

Ossenkoppele et al., "Tau PET correlates with different Alzheimer's disease related features than CSF and plasma biomarkers of tau pathology".

Content:

Table	Title	Page
S1	Percentage of β-coefficient change between the simple and combined model in BioFINDER-2	2
S2	Difference of R-squared between ridge regression models for tau PET vs CSF p-tau181 in BioFINDER-2	3
S3	Proportion of missing values	4

Table S1. Percentage of β-coefficient change between the simple and combined model in BioFINDER-2

	**Total**			**CU**			**MCI/Dem**		
**Feature**	**PET**	**CSF**		**PET**	**CSF**		**PET**	**CSF**	
Age	-86<br>(-147, -57)	**-8**<br>**(-36, 20)**	*	-75<br>(-88, -60)	-58<br>(-80, -35)		-83<br>(-139353, 162532)	48<br>(-96, 10655)	
APOE ε4 carriership	-53<br>(-127, -34)	-35<br>(-55, 16)		-68<br>(-105110, 15928)	-8<br>(-47, 56)		-38<br>(-87, 56)	-42<br>(-97, 214)	
CSF A$\beta_{42/40}$	-85<br>(-110, -67)	**-8**<br>**(-20, 6)**	*	-92<br>(-143, -71)	**-14**<br>**(-38, 6)**	*	-71<br>(-105, -52)	**-14**<br>**(-28, 4)**	*
Amyloid PET SUVR	-61<br>(-72, -50)	**-31**<br>**(-42, -21)**	*	-64<br>(-76, -52)	-29<br>(-53, -14)		-57<br>(-70, -41)	-38<br>(-49, -26)	
MMSE	**-4**<br>**(-19, 7)**	-92<br>(-115, -70)	*	-81<br>(-159, 59)	-58<br>(-82, -22)		**8**<br>**(-8, 24)**	-118<br>(-176, -80)	*
Memory composite	**-18**<br>**(-34, -6)**	-69<br>(-87, -52)	*	-78<br>(-466, 343)	-31<br>(-97, 29)		**1**<br>**(-16, 18)**	-101<br>(-156, -71)	*
Language composite	**-22**<br>**(-42, 0)**	-65<br>(-100, -44)	*	-71<br>(-91, -48)	-51<br>(-71, -32)		-11<br>(-42, 27)	-74<br>(-199, -41)	
Executive functioning composite	**-19**<br>**(-37, -1)**	-68<br>(-96, -50)	*	-65<br>(-122, -30)	-39<br>(-69, -14)		**-3**<br>**(-36, 31)**	-89<br>(-220, -46)	*
Visuospatial composite	**6**<br>**(-38, 38)**	-114<br>(-232, -55)	*	-32<br>(-99, 131)	-27<br>(-1360, 1175)		**12**<br>**(-36, 60)**	-133<br>(-2729, -47)	*
MRI Hippocampal volume/ TIV ratio	-17<br>(-44, 7)	-72<br>(-116, -40)		-20<br>(-263, 198)	-6<br>(-894, 724)		-15<br>(-63, 29)	-69<br>(-203, -18)	
MRI AD signature cortical thickness	**4**<br>**(-11, 18)**	-108<br>(-140, -80)	*	-44<br>(-79, 1)	-67<br>(-252, 52)		**22**<br>**(5, 44)**	-172<br>(-292, -112)	*

Data are presented as the median (95% confidence interval [CI]) of the change in percentage of the β-coefficient between the simple and the combined model for both tau PET and CSF ptau-181. Values were taken from the ridge regression models over 1000 bootstrapped samples. Asterisks and bold text mark the cases where the 95% CI between temporal meta-ROI tau PET and CSF p-tau181 models did not overlap, indicating that there is a greater association between the patient feature and the modality that has a smaller decrease in β-coefficient.

Table S2. Difference of R-squared between ridge regression models for tau PET vs CSF p-tau181 in BioFINDER-2

	**Total**			**CU**			**MCI / Dem**		
**Feature**	**PET**	**CSF**		**PET**			**PET**	**CSF**	
Age	0.06 (0.03, 0.10)	0.11 (0.07, 0.16)		0.14 (0.09, 0.23)	0.16 (0.10, 0.23)		0.02 (0.00, 0.08)	0.04 (0.00, 0.12)	
CSF $A\beta_{42/40}$	0.37 (0.31, 0.43)	**0.50 (0.45, 0.55)**	*	0.22 (0.14, 0.30)	**0.47 (0.38, 0.55)**	*	0.29 (0.19, 0.39)	0.41 (0.32, 0.49)	
Amyloid PET SUVR	0.47 (0.39, 0.55)	0.57 (0.49, 0.65)		0.39 (0.25, 0.51)	0.61 (0.49, 0.70)		0.47 (0.34, 0.62)	0.47 (0.35, 0.62)	
MMSE	**0.59 (0.51, 0.68)**	0.36 (0.28, 0.44)	*	0.08 (0.02, 0.17)	0.09 (0.03, 0.18)		**0.54 (0.43, 0.64)**	0.23 (0.13, 0.34)	*
Memory composite	0.55 (0.48, 0.61)	0.45 (0.38, 0.51)		0.30 (0.21, 0.40)	0.31 (0.21, 0.40)		0.51 (0.41, 0.60)	0.30 (0.17, 0.41)	
Language composite	0.46 (0.37, 0.54)	0.38 (0.29, 0.46)		0.25 (0.17, 0.34)	0.25 (0.17, 0.34)		0.32 (0.19, 0.44)	0.20 (0.09, 0.33)	
Executive functioning composite	0.48 (0.40, 0.55)	0.39 (0.32, 0.47)		0.39 (0.29, 0.48)	0.39 (0.30, 0.49)		0.25 (0.14, 0.35)	0.13 (0.05, 0.22)	
Visuospatial composite	0.30 (0.19, 0.43)	0.15 (0.07, 0.24)		0.08 (0.02, 0.16)	0.07 (0.01, 0.14)		0.23 (0.12, 0.36)	0.08 (0.00, 0.18)	
MRI Hippocampal volume/ TIV ratio	0.42 (0.36, 0.49)	0.39 (0.33, 0.46)		0.37 (0.28, 0.47)	0.37 (0.28, 0.47)		0.27 (0.17, 0.39)	0.24 (0.14, 0.34)	
MRI AD signature region thickness	**0.52 (0.46, 0.58)**	0.39 (0.33, 0.45)	*	0.36 (0.23, 0.50)	0.28 (0.19, 0.39)		0.46 (0.35, 0.56)	0.27 (0.16, 0.39)	

Data are presented as the median (95% confidence interval [CI]) of R-squared values from simple models for tau PET in the temporal meta-ROI and CSF p-tau181. Values were taken from the ridge regression models over 1000 bootstrapped samples. Asterisks and mark cases where the 95% CI between PET and CSF models do not overlap, indicating that there is a greater association between the patient feature and the modality that has the bigger R-squared value (also marked by bold).

Table S3. Proportion of missing values

	BioFINDER-2 (n=404)	ADNI (n=371)
Education, years	1 (0.2)	0 (0)
APOE ε4 carriership	0 (0)	0 (0)
Plasma p-tau181	1 (0.2)	-
Plasma p-tau217	1 (0.2)	-
CSF p-tau181	0 (0)	0 (0)
CSF p-tau217	0 (0)	
CSF t-tau		0 (0)
Tau PET	0 (0)	0 (0)
CSF $A\beta_{42/40}$	0 (0)	33 (8.9)
Amyloid PET	83 (20.8)	1 (0.3)
MMSE	0 (0)	0 (0)
Memory composite	11 (2.8)	1 (0.3)
Language composite	7 (1.8)	0 (0)
Executive functioning composite	3 (0.8)	0 (0)
Visuospatial composite	20 (5.0)	0 (0)
MRI Hippocampal volume/ TIV ratio	9 (2.2)	7 (1.9)
MRI AD signature region thickness	9 (2.2)	17 (4.6)

Missing values are presented as n (%)

TIV = Total intracranial volume.
