# Supplementary material for: Tau PET correlates with different Alzheimer’s disease‐related features compared to CSF and plasma p‐tau biomarkers
Source: EMBO Mol Med. 2021 Jul 13;13(8):e14398. doi: 10.15252/emmm.202114398 (PMC8350902; doi:10.15252/emmm.202114398)
Supplement: Supplementary file 2 — Expanded View Figures PDF [file EMMM-13-e14398-s001.pdf]

Expanded View Figures

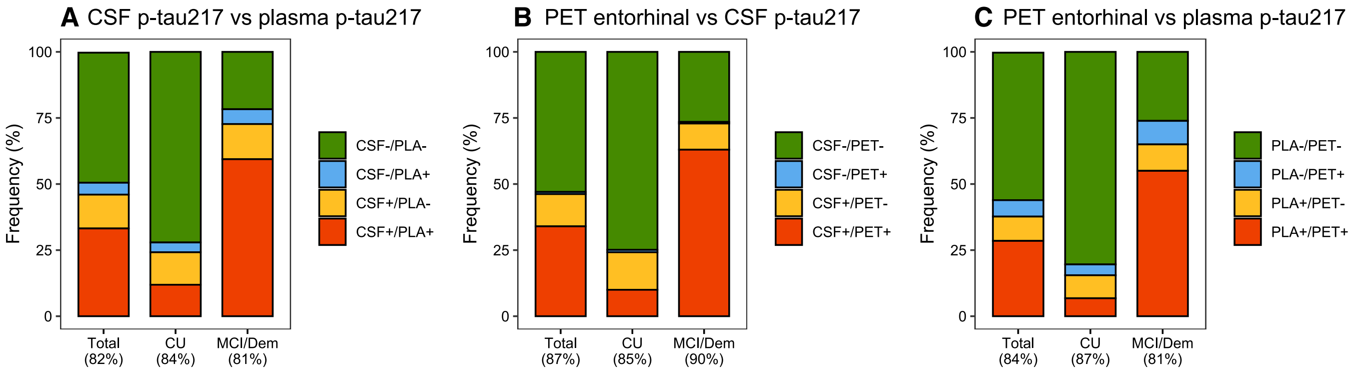

Figure EV1. Concordance between tau PET in the entorhinal cortex, CSF ptau-217 and plasma p-tau217 in BioFINDER-2.

A–C The graphs represent concordance rates between different tau biomarkers in BioFINDER-2: (A) CSF p-tau217 vs plasma p-tau217, (B) Tau PET in the entorhinal cortex vs CSF p-tau217 and (C) Tau PET in the entorhinal cortex vs plasma p-tau217. Cut-offs for both tau PET and fluid biomarkers are based on the mean + (2 × standard deviation) in Aβ-negative cognitively normal individuals (see Materials and Methods section for further detail).

Figure EV2. Differential associations of tau PET vs CSF p-tau181 with Alzheimer-related features by amyloid status. ▶

Graphs display the differential association of temporal Meta-ROI tau PET vs CSF p-tau181 with AD-related features in the BioFINDER-2 study stratified by amyloid status in the total population and in CU. Median and 95% confidence intervals (CI) of β-coefficients are plotted from the following ridge regression models: (i) AD-related feature predicted by CSF p-tau181 (dark blue, simple model), (ii) AD-related feature predicted by tau PET (dark red, simple model), (iii) AD-related feature predicted by the combination of CSF p-tau181 (light blue) and tau PET (orange, combined model). In case a feature was non-significant for both tau biomarkers in the simple models (i.e. 95% CIs crossed the 0-line), no combined model was performed. All models were adjusted for age and sex, and cognitive tests were additionally adjusted for education. The β-coefficients for age, *APOE* ε4 carriership and amyloid PET global measures were multiplied by −1 for visualization purposes. In amyloid-negative CU, β-coefficients for models for APOE ε4 status are unavailable, as bootstrapping resulted in unbalanced samples. To compare the strength of the associations between PET and CSF tau biomarkers with the predicted AD-features, we followed these three criteria: (i) non-overlapping 95% CIs of the β-coefficient of the simple models for CSF and PET (i.e. stronger association for the biomarker with the more positive or negative value), (ii) 95% CIs in the simple or combined models non-overlapping with β = 0 for only CSF or PET (stronger association for the tau biomarker non-overlapping with β = 0), (iii) a significant drop of the β-coefficient from the combined model relative to the simple model for only CSF or PET (i.e. overlapping 95% CIs for one tau biomarker between the simple and combined model, but non-overlapping 95% CIs for the other, with a stronger association for the biomarker with overlapping 95% CIs).

 © 2021 The Authors

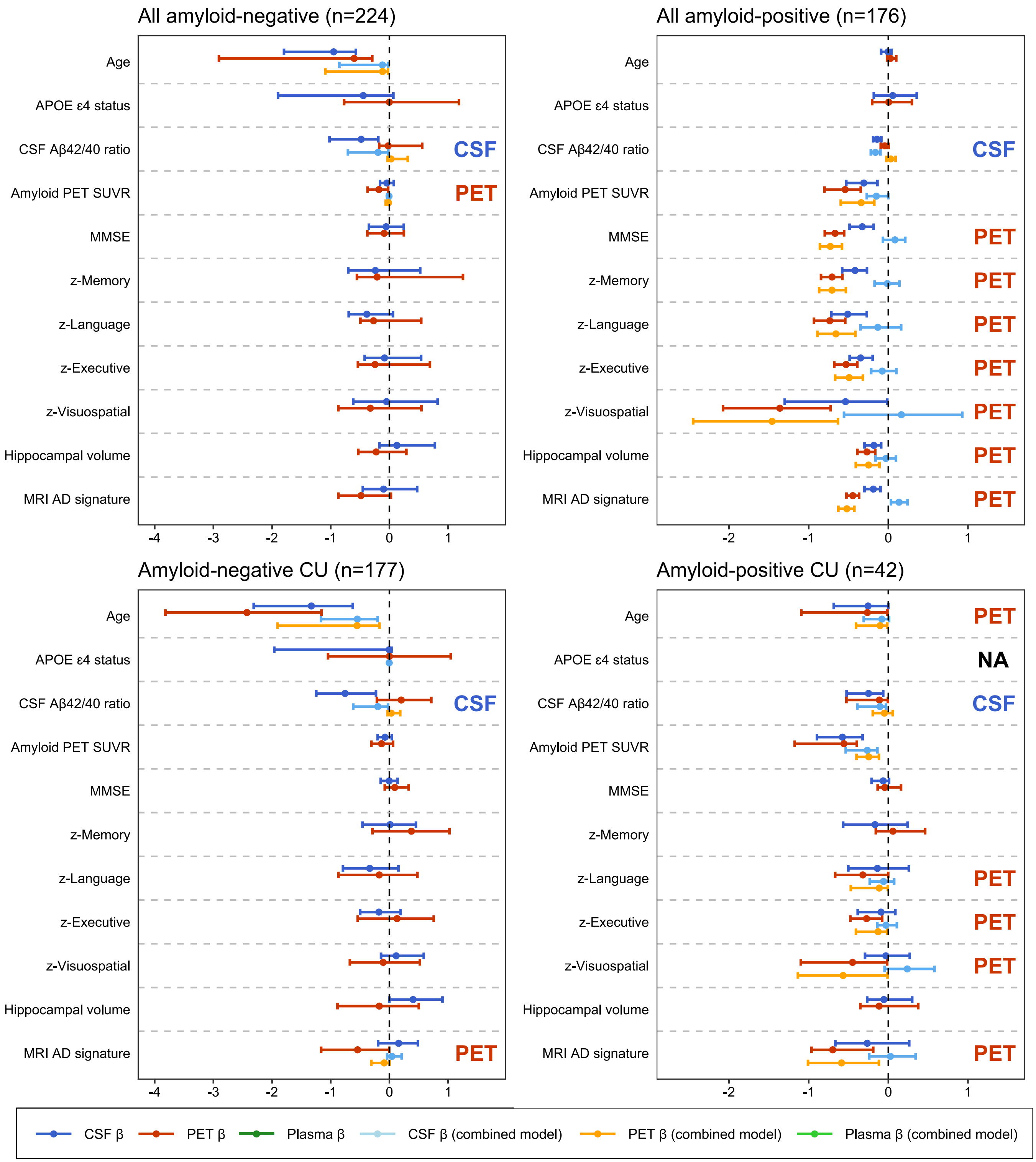
All amyloid-negative (n=224)
All amyloid-positive (n=176)
Amyloid-negative CU (n=177)
Amyloid-positive CU (n=42)
Age
APOE ε4 status
CSF Aβ42/40 ratio
Amyloid PET SUVR
MMSE
z-Memory
z-Language
z-Executive
z-Visuospatial
Hippocampal volume
MRI AD signature
CSF
PET
NA
-4
-3
-2
-1
0
1
CSF β
PET β
Plasma β
CSF β (combined model)
PET β (combined model)
Plasma β (combined model)

Figure EV2.

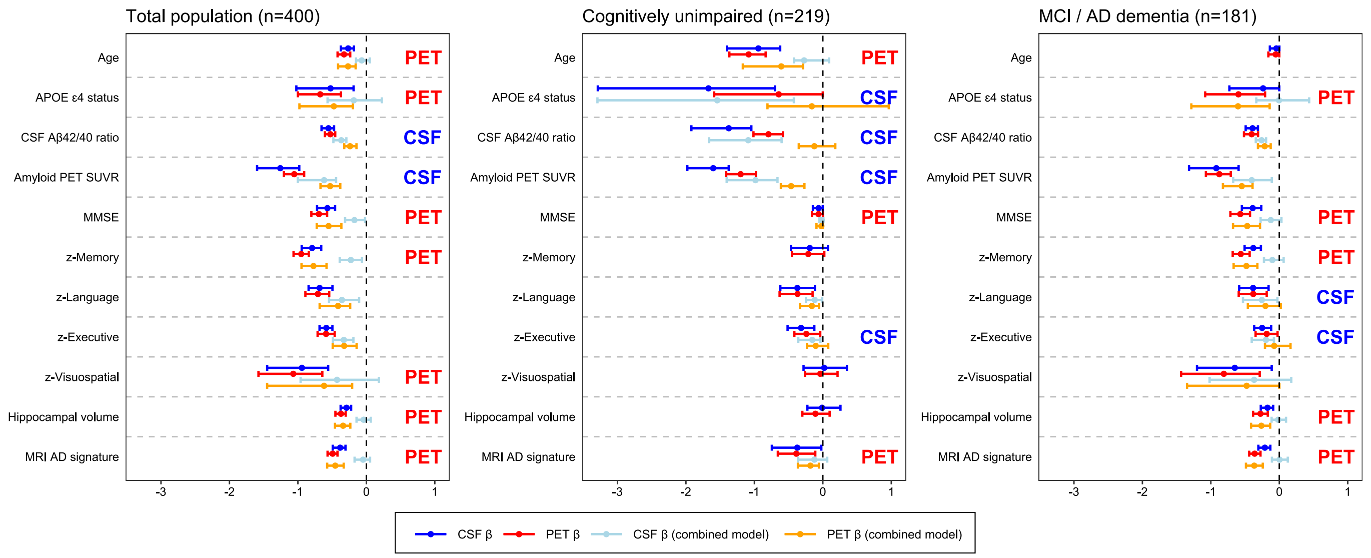

Figure EV3. Differential associations of entorhinal cortex tau PET vs CSF p-tau217 with Alzheimer-related features in BioFINDER-2.

Graphs display the differential association of entorhinal cortex tau PET vs CSF p-tau217 with AD-related features in BioFINDER-2. Median and 95% confidence intervals (CI) of β-coefficients are plotted from the following ridge regression models: (i) AD-related feature predicted by CSF p-tau217 (dark blue, simple model), (ii) AD-related feature predicted by tau PET (dark red, simple model), (iii) AD-related feature predicted by the combination of CSF p-tau217 (light blue) and tau PET (orange, combined model). In case a feature was non-significant for both tau biomarkers in the simple models (i.e. 95% CIs crossed the 0-line), no combined model was performed. All models were adjusted for age and sex, and cognitive tests were additionally adjusted for education. The β-coefficients for age, *APOE* ε4 carriership and amyloid PET global measures were multiplied by −1 for visualization purposes.

To compare the strength of the associations between PET and CSF tau biomarkers with the predicted AD-features, we followed these three criteria: (i) non-overlapping 95% CIs of the β-coefficient of the simple models for CSF and PET (i.e. stronger association for the biomarker with the more positive or negative value), (ii) 95% CIs in the simple or combined models non-overlapping with $\beta = 0$ for only CSF or PET (stronger association for the tau biomarker non-overlapping with $\beta = 0$), (iii) a significant drop of the β-coefficient from the combined model relative to the simple model for only CSF or PET (i.e. overlapping 95% CIs for one tau biomarker between the simple and combined model, but non-overlapping 95% CIs for the other, with a stronger association for the biomarker with overlapping 95% CIs).

© 2021 The Authors

The main analysis was performed using ridge regression using the *glmnet* library in R. We chose to use ridge regression because it provides stable estimates of β-coefficients despite correlated predictors, which is the case in our combined models. Compared to ordinary least square regression, ridge regression additionally uses L2 regularisation to penalise residuals to minimize both loss and complexity of the model. The resulted model typically fits the data less well than ordinary least square model, but is more generalizable to other data, as it is less sensitive to variance such as outliers.

To compare the β-coefficients between models we used bootstrapped sampling with replacement (N=1,000 iterations). The alpha value was set as 0 to get ridge regression as opposed to lasso regression. For each model in a bootstrapped sample, we tested an different regularisation rates (lambda) using the cv.glmnet() function using a predefined set of N=40 values from 0.01 to 100. This function uses cross-validation to test the mean-squared error of the models and results in an optimal lambda value that best minimises the error in cross-validation. This lambda value was then used in the final model for that bootstrapped sample to get β-coefficients. As *APOE* ε4 status is a binary variable, a binomial regression was used.

This resulted in three models per bootstrapped sample, shown below with the example of MMSE in the main analysis comparing Tau PET temporal Meta-ROI and CSF p-tau181:

1) Simple PET model: MMSE <- **Tau PET temporal Meta-ROI +** Age + Sex + Education

2) Simple CSF model: MMSE <- **CSF p-tau181** + Age + Sex + Education

3) Combined CSF model: MMSE <- **Tau PET temporal Meta-ROI** + **CSF p-tau181** + Age + Sex + education

Then using N=1000 bootstrapped samples, we computed 95% confidence intervals (CI) of the four β-coefficients (shown as bold above): a) PET from the simple model, b) CSF from the simple model, c) PET from the combined model, and d) CSF from the combined model.

To compare the strength of the associations between PET and CSF tau biomarkers with the predicted AD-features, we followed three criteria:

(i) Non-overlapping 95% CIs of the β-coefficient of the simple models for CSF and PET (i.e., stronger association for the biomarker with the more positive or negative value). An example of this is amyloid PET SUVR having a higher association to Tau PET temporal Meta-ROI compared to plasma p-tau181 in the total population of BioFINDER-2.

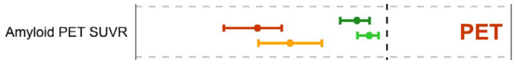

(ii) 95 % CIs in the simple or combined models non-overlapping with β=0 for only CSF or PET (stronger association for the tau biomarker non-overlapping with β=0). Examples for that include MMSE having a higher association to Tau PET temporal Meta-ROI compared to CSF p-tau 181 in the total population in BioFINDER-2 (in the combined model the CI for CSF overlaps β=0, whereas the CI for PET is non-overlapping) and MRI AD signature having a higher association to PET than CSF in the cognitively unimpaired (CU) in BioFINDER-2 (both the CIs from the simple and combined models for CSF overlap β=0, whereas the coefficients for PET do not).

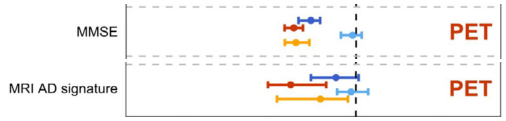

(iii) A significant drop of the β-coefficient from the combined model relative to the simple model for only CSF or PET (i.e., overlapping 95% CIs for one tau biomarker between the simple and combined model, but non-overlapping 95% CIs for the other, with a stronger association for the biomarker with overlapping 95% CIs). An example for this is amyloid PET SUVR having a stronger association to CSF p-tau181 compared to Tau PET temporal meta-ROI in the total population in BioFINDER-2 (the CIs of the simple and combined model overlap for CSF, but not for PET).

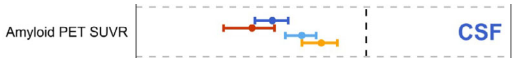

Figure EV4. Schematic overview of the Ridge regression model approach.
