# Supplementary material for: Tau PET correlates with different Alzheimer’s disease‐related features compared to CSF and plasma p‐tau biomarkers
Source: EMBO Mol Med. 2021 Jul 13;13(8):e14398. doi: 10.15252/emmm.202114398 (PMC8350902; doi:10.15252/emmm.202114398)
Supplement: Supplementary file 3 — Table EV1 [file EMMM-13-e14398-s002.docx]

**Table EV1**. R-squared values for simple models for CSF p-tau181 and CSF p-tau217 in BioFINDER-2

|  | **Total** |  |  | **CU** |  |  | **MCI / Dem** |  |
| --- | --- | --- | --- | --- | --- | --- | --- | --- |
| **Feature** | **CSF p-tau181** | **CSF p-tau217** |  | **CSF p-tau181** | **CSF p-tau217** |  | **CSF p-tau181** | **CSF p-tau217** |
| Age | 0.11 (0.07, 0.16) | 0.09 (0.05, 0.13) |  | 0.16 (0.10, 0.23) | 0.12 (0.07, 0.19) |  | 0.04 (0.00, 0.12) | 0.03 (0.00, 0.10) |
| CSF Aβ_42/40_ | 0.50 (0.45, 0.55) | 0.49 (0.44, 0.54) |  | 0.47 (0.38, 0.55) | 0.49 (0.42, 0.58) |  | 0.41 (0.32, 0.49) | 0.40 (0.32, 0.48) |
| Amyloid PET SUVR | 0.57 (0.49, 0.65) | 0.62 (0.52, 0.71) |  | 0.61 (0.49, 0.70) | 0.71 (0.62, 0.77) |  | 0.47 (0.35, 0.62) | 0.49 (0.35, 0.65) |
| MMSE | 0.36 (0.28, 0.44) | 0.40 (0.33, 0.48) |  | 0.09 (0.03, 0.18) | 0.09 (0.03, 0.18) |  | 0.23 (0.13, 0.34) | 0.26 (0.16, 0.37) |
| Memory composite | 0.45 (0.38, 0.51) | 0.48 (0.41, 0.54) |  | 0.31 (0.21, 0.40) | 0.30 (0.22, 0.40) |  | 0.30 (0.17, 0.41) | 0.32 (0.20, 0.44) |
| Language composite | 0.38 (0.29, 0.46) | 0.40 (0.30, 0.48) |  | 0.25 (0.17, 0.34) | 0.25 (0.17, 0.35) |  | 0.20 (0.09, 0.33) | 0.21 (0.09, 0.34) |
| Executive functioning composite | 0.39 (0.32, 0.47) | 0.42 (0.35, 0.50) |  | 0.39 (0.30, 0.49) | 0.40 (0.31, 0.49) |  | 0.13 (0.05, 0.22) | 0.15 (0.06, 0.25) |
| Visuospatial composite | 0.15 (0.07, 0.24) | 0.17 (0.09, 0.29) |  | 0.07 (0.01, 0.14) | 0.06 (0.01, 0.14) |  | 0.08 (0.00, 0.18) | 0.10 (0.02, 0.22) |
| MRI Hippocampal volume/ TIV ratio | 0.39 (0.33, 0.46) | 0.41 (0.35, 0.47) |  | 0.37 (0.28, 0.47) | 0.37 (0.28, 0.47) |  | 0.24 (0.14, 0.34) | 0.25 (0.15, 0.36) |
| MRI AD signature region thickness | 0.39 (0.33, 0.45) | 0.42 (0.36, 0.48) |  | 0.28 (0.19, 0.39) | 0.30 (0.20, 0.43) |  | 0.27 (0.16, 0.39) | 0.29 (0.19, 0.41) |

Data are presented as the median (95% confidence interval [CI]) of R-squared values for the simple models for CSF p-tau181 and CSF p-tau217. Values were taken from the ridge regression models over 1000 bootstrapped samples.
